# Endocrine and Transcriptome Changes Associated with Testicular Growth and Differentiation in Atlantic Salmon (*Salmo salar* L.)

**DOI:** 10.3390/cimb46060319

**Published:** 2024-05-27

**Authors:** Vetle Skjold, Sergey Afanasyev, Erik Burgerhout, Lene Sveen, Kjell-Arne Rørvik, Vasco Felipe Cardoso Neves Mota, Jens-Erik Dessen, Aleksei Krasnov

**Affiliations:** 1 The Norwegian Institute of Aquaculture, Nofima, 9291 Tromsø, Norway; vetle.skjold@nofima.no (V.S.); erik.burgerhout@nofima.no (E.B.); lene.sveen@nofima.no (L.S.); kjell-arne.rorvik@nofima.no (K.-A.R.); jens-erik.dessen@nofima.no (J.-E.D.); 2 Department of Mechanical Engineering and Technology Management, Norwegian University of Life Sciences, 1433 Ås, Norway; vasco.mota@nmbu.no; 3 Sechenov Institute of Evolutionary Physiology and Biochemistry, 194223 Saint Petersburg, Russia; afanserg@mail.ru

**Keywords:** Atlantic salmon, testes, pituitary, sex steroids, transcriptome

## Abstract

Sexual maturation of Atlantic salmon males is marked by dramatic endocrine changes and rapid growth of the testes, resulting in an increase in the gonad somatic index (GSI). We examined the association of gonadal growth with serum sex steroids, as well as pituitary and testicular gene expression levels, which were assessed with a DNA oligonucleotide microarray. The testes transcriptome was stable in males with a GSI < 0.08% despite the large difference between the smallest and the largest gonads. Fish with a GSI ≥ 0.23% had 7–17 times higher serum levels of five male steroids and a 2-fold increase in progesterone, without a change in cortisol and related steroids. The pituitary transcriptome showed an upregulation of the hormone-coding genes that control reproduction and behavior, and structural rearrangement was indicated by the genes involved in synaptic transmission and the differentiation of neurons. The observed changes in the abundance of testicular transcripts were caused by the regulation of transcription and/or disproportional growth, with a greater increase in the germinative compartment. As these factors could not be separated, the transcriptome results are presented as higher or lower specific activities (HSA and LSA). LSA was observed in 4268 genes, including many genes involved in various immune responses and developmental processes. LSA also included genes with roles in female reproduction, germinal cell maintenance and gonad development, responses to endocrine and neural regulation, and the biosynthesis of sex steroids. Two functional groups prevailed among HSA: structure and activity of the cilia (95 genes) and meiosis (34 genes). The puberty of A. salmon testis is marked by the predominance of spermatogenesis, which displaces other processes; masculinization; and the weakening of external regulation. Results confirmed the known roles of many genes involved in reproduction and pointed to uncharacterized genes that deserve attention as possible regulators of sexual maturation.

## 1. Introduction

The structure and development of teleost testes have been described in several excellent reviews [1,2,3,4]. In short, progenitor gonads are formed in males and females as two stretches, which are then populated by germinal cells (GC). The development of GC includes two critical decisions: sexual commitment for sperm or egg production and the transition from maintaining mitotic division to entering meiosis, which can occur as single or separate events [5]. Sex determination in salmonid GC is not irreversible. Transplanted rainbow trout testicular germ cells differentiate into spermatozoa and fully functional eggs depending on the recipient’s sex [6]. The testis consists of two compartments. The interstitial (intertubular) compartment contains hormone-producing Leydig cells, blood vessels, macrophages and other immune cells, and neural and connective tissue elements, including peritubular myoid cells. The germinative compartment (spermatogenic) contains only Sertoli cells and cysts with clonal GC, each derived from a single spermatogonia stem cell. The onset of terminal spermatogenesis in male gonads is marked by the appearance of cystic structures containing type B spermatogonia, which continue synchronous mitotic divisions [7]. The subsequent stages of spermatogenesis are primary and secondary meiotic spermatocytes, haploid spermatids, and spermatozoa. The main events of spermiogenesis are DNA condensation, elimination of organelles and cytoplasm, and the formation of cilia (flagella). Sexual maturation is stimulated by elevated levels of pituitary and steroid sex hormones, leading to rapid growth and changes in the structure of the testes. A panel of regulators has been identified, which includes hormones and genes with conserved functions across vertebrates, as well as those present only in teleosts.

Precocious maturation in commercial salmon farming is highly unwanted yet frequently reported both in commercial production at sea and in post-smolt production in closed containment systems (CCS). The protocols used in CCS usually involve continuous light and elevated water temperature, factors known to increase the risk of early initiation of maturation [8,9]. Maturation leads to poor growth and the degrading of the product quality, resulting in large economic losses [10,11]. Another problem is farmed escapees threatening wild populations, and the sterilization of Atlantic salmon is a priority for genetic manipulation. Knowledge of the genetic, molecular, and cellular mechanisms of testes development is essential for diagnosis and control of sexual maturation in aquaculture. The production of sterile triploids was introduced into aquaculture several decades ago [12,13]. Current research is focused on silencing genes that play key roles in germ cell maintenance and sperm development, such as *fsh* and *dnd* [14,15,16,17]. Transcriptomics provides a powerful approach to understanding developmental processes and the discovery of genes, which can serve as diagnostic markers and targets for manipulation [14,18]. Microarrays and next-generation sequencing analyses have addressed a variety of research tasks, such as molecular markers for type A and B spermatogonia [19,20], comparison of spermatogonia and testicular somatic cells [21], sex reversal [22], in vitro responses of rainbow trout testes to gonadotropins [23], and others. Here, we examined the changes associated with sexual maturity indicated by endocrine changes and accelerated testicular growth. Combining new results with a meta-analysis of the Atlantic salmon transcriptome database [24], we evaluated the development trajectories of gonad-specific genes.

## 2. Materials and Methods

### 2.1. Ethical Statement

The experiment took place at the Nofima Research Station for Sustainable Aquaculture in Sunndalsøra, Norway. These research facilities possess licenses according to Norwegian law, enabling them to conduct experimental work on fish in accordance with regulation FOR-2015-06-18-761 “Forskrift om bruk av dyr i forsøk”. This regulation aligns nationally with and adheres to the “Directive 2010/63/EU of the European Parliament and of the Council on the protection of animals used for scientific purposes”. Samplings were conducted on euthanized fish, thereby falling within the scope of the exemption provided by the aforementioned regulation, specifically §2 article f. This section states that a specific license is unnecessary when experimental treatments are not anticipated to result in any detrimental effects on animals. Tagging of the fish in the experiment was performed under the general tagging license of the site, specifically FOTS ID 29627 (https://www.mattilsynet.no/dyr/forsoksdyr/soknader, accessed on 12 September 2023).

### 2.2. Fish Material and Rearing Conditions

The fish material utilized in the current study was obtained from an experiment described in detail elsewhere [25]. The farmed Atlantic salmon post-smolts were from the breeding nucleus of AquaGen AS (QTL, IPN/PD/CMS/LICE), originally sources form from the commercial freshwater facility Belsvik of Lerøy Midt AS (Hellandsjøen, Mid-Norway). Before the transport and commencement of the experiment at Nofima Research Station for Sustainable Aquaculture, all salmon underwent routine vaccination against red mouth disease (bath with AlphaDIP ERM, Pharmaq, Norway), *Aeromonas salmonicida*, *Listonella anguillarum*, *Moritella viscosa*, infectious pancreatic necrosis (intraperitoneal injection with the oil-adjuvanted vaccine AlphaJECT micro 6, Pharmaq), and salmon alphavirus (intramuscular injection with the DNA-vaccine Clynav, Elanco, Norway). The salmon were smoltified according to standard procedures with an induced “winter signal” (light–darkness [L:D] 24:0–12:12–24:0), and detailed information regarding the experimental setup, diets, and fish husbandry can be found in [25].

### 2.3. Sampling

The fish analyzed in the current study were sampled after 18 weeks’ rearing in a brackish water flow-through system, with an average salinity of 20 ± 0.4 ppt and mean temperature of 12.1 ± 0.2 °C. The salmon were gently netted from the tanks and subsequently anesthetized using MS-222 (Metacaine 0.1 g L^−1^; Alpharma, Animal Health Ltd., Hampshire, UK) dissolved in oxygenated water. After anesthetization, several measurements were recorded, including passive integrated transponder tag (PIT-tag) data, weight, and length. Blood samples were drawn from the caudal vein, using vacutainers (BD Vacutainer^®^ with silica clot activator, an interior coated with silicone, and a red BD Hemogard™ closure). After a coagulation time of 30 min, the blood was centrifuged at relative centrifugal force of 2200× *g* for 10 min, and serum was frozen at −80 °C. Following the initial procedures, the fish were killed using a lethal dose of MS-222. Afterwards, the fish were dissected to determine their sex, and subsequently, the pituitary gland and testis were carefully extracted. After weighing the testis, one part of the testis and the entire pituitary gland were stored in RNAlater™ (Invitrogen, Thermo Fisher Scientific, Waltham, MA, USA). Residual testis was placed in 10% neutral formalin containers (BiopSafe^®^, Mermaid Medical, Stenløse, Denmark) for histological analysis.

### 2.4. Testis Histology and Blood Steroid Hormones

Testis histology was studied as described in [25]. Testis scoring in the current study used a two-step approach, including assessment of the most advanced germ cells and the predominant germ cells within the tissue. This approach was necessitated by the presence of multiple maturation stages across the section in several samples. The size of the germinative compartments was also assessed, and observations of interest were noted. The serum content of the analytes 17a-OH-progesterone, testosterone, progesterone, 21-deoxycortisol, 11-deoxycortisol, corticosterone, cortisol, androstenedione, 4-androsten-11B-OL3-17-dione, 4-androsten-3–11-17-trione (andrenosterone), and 4-androsten-17B-OL3-11B-dione (11-ketotestosterone) were measured via liquid chromatography tandem mass spectrometry at the Proteomics and Metabolomics Core Facility (PRiME, Tromsø, Norway) [25].

### 2.5. Microarray

Total RNA was extracted from the testis and pituitary. Tissue pieces were placed in tubes with steel beads containing 400 µL lysis buffer (Qiagen, Hilden, Germany) and 20 µL proteinase K (50 mg/mL), homogenized in FastPrep-96 (MP Biomedicals, Santa Ana, CA, USA) for 120 s at maximum speed, centrifuged, and incubated for 30 min at 37 °C. RNA was extracted on Biomek 4000 robot using the Agencourt RNAdvance Tissue kit including an on-column DNase treatment (Qiagen). The purity and integrity of the RNA samples (RIN values > 7.5) were checked using a NanoDrop ND-1000 Spectrophotometer (NanoDrop Technologies, Wilmington, DE, USA) and 2100 Bioanalyzer with RNA Nano Chips (Agilent Technologies, Santa Clara, CA, USA). Nofima’s 44 K Atlantic salmon oligonucleotide microarray Salgeno-2 (GPL28080) was fabricated by Agilent Technologies; reagents and equipment were purchased from the same provider. RNA was amplified, labeled, and fragmented using a One-Color Quick Amp Labeling Kit and a Gene Expression Hybridization kit. The total RNA input for each reaction was 500 ng. After overnight hybridization in an oven (17 h, 65 °C, rotation speed 0.01× *g*), the arrays were washed with Gene Expression Wash Buffers 1 and 2 and scanned with SureScan, Agilent Technologies. Nofima’s bioinformatic system STARS [26] was used for data analyses.

### 2.6. Calculations

Gonadosomatic Index (GSI, %) was calculated as

(1) $$\mathrm{GSI}=\frac{\mathrm{Weight}\;\mathrm{gonad}}{\mathrm{Body}\;\mathrm{weight}}\times 100.$$

Condition factor (CF) was calculated as

(2) $$\mathrm{CF}=\frac{W}{L^{3}}\times 100,$$

where W is fish weight in g, and L is fork length in cm.

Differences between immature and pubertal test were assessed via *t*-test. The Pearson product–moment correlation coefficient was used to describe the association between two variables. The level of significance was chosen at *p* ≤ 0.05. Differential gene expression (HAS and LSA) was determined via the criteria: log_2_-ER (expression ratio) > 0.8 (1.75-fold) and *p* < 0.05; complete results are in Supplementary Table S1. Functional groups of genes (STARS annotation) with coordinated expression differences were identified via significant deviation of mean log_2_-ER from zero. The gonad-specific genes (GSG) were searched using meta-analyses of Nofima’s microarray database [26]. GSG expression in testes and/or ovaries but not in any other tissue was eight-fold higher than the average of all tissues.

## 3. Results

### 3.1. Biometrics, Morphology, and Testis Histology

**Figure 1:**
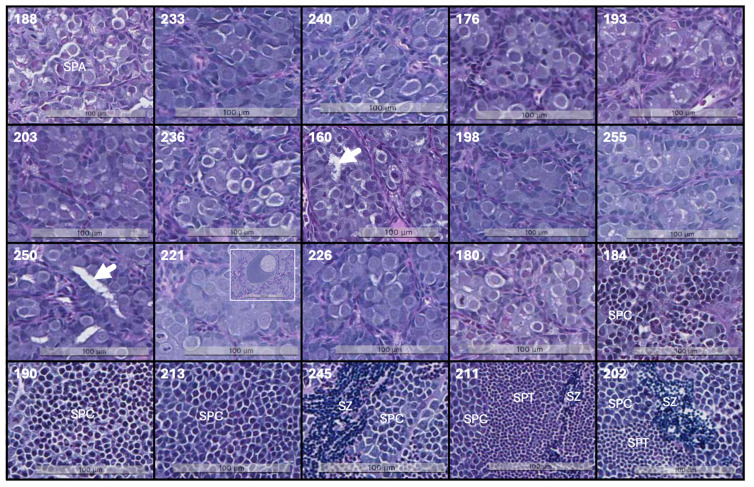
Histology of the testis. In fish 188–180, spermatogonia A (SPA) predominate in compact germinal compartments. Fish 160 and 250 exhibit tubules within the germinal compartment (highlighted by arrows). The small insert in 221 displays oocyte in the testis. Fish 190–202 show more advanced cell stages characterized by a reduction in nucleus diameter and darker staining of the nucleus due to condensed genetic material; spermatocytes (SPC), spermatids (SPT), and spermatozoa (SPZ) are indicated. The scale bar shown in the images indicates size. Fish are organized by ascending GSI. Sample identity, denoted by the number in the upper left corner, corresponds to Table 1. The samples were qualified as in Skjold et al. [25]. Tissue sections were stained with alcian blue and periodic acid Schiff.

The twenty post-smolt males used in this study had an average body weight, length, and condition factor of 1017 ± 82 g, 41.3 ± 0.8 cm, and 1.44 ± 0.08, respectively (mean ± standard deviation, SD). There were no differences associated with GSI development. Six of the twenty individual fish with GSI ≥ 0.23% showed advanced testis development characterized by the presence of spermatogonia type B cells, spermatocytes, spermatids, and spermatozoa (Table 1, Figure 1). Morphological changes associated with maturation were measured on all fish as described by Skjold et al. [25]. Five out of six individuals with GSI ≥ 0.23% had developed skin spots associated with early maturity. Two out of fourteen individuals with GSI < 0.08% had increased germinal compartments with clearly defined tubules and active cell division in the A-spermatogonia cyst, but GSI were within the range characteristic of immature testes (0.023% and 0.031%). Oocytes were found in the testes of three individuals.

### 3.2. Serum Hormones and Pituitary Gene Expression

**Figure 2:**
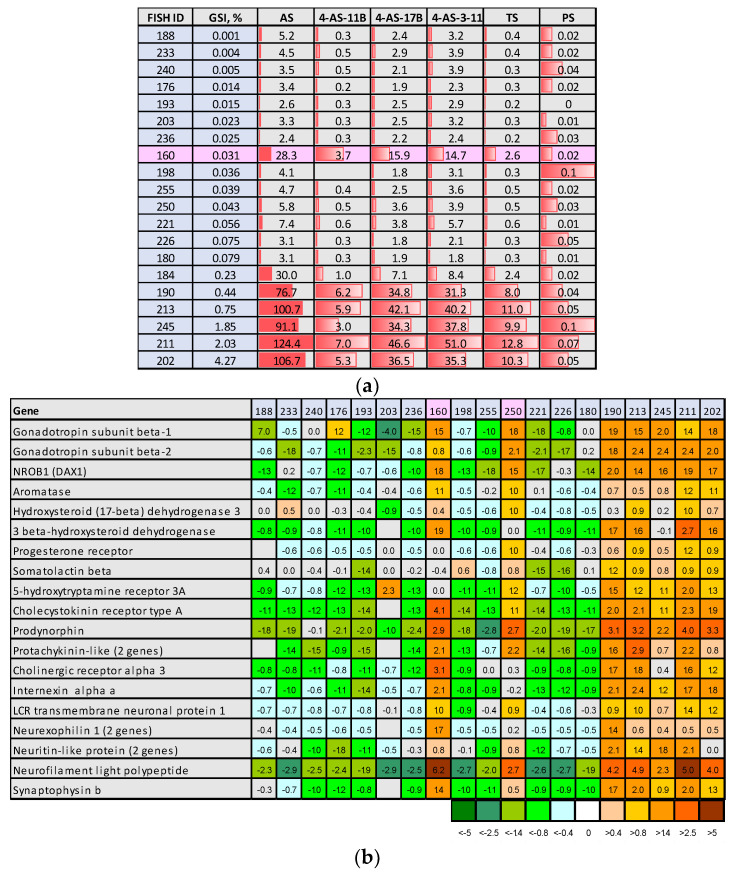
Endocrine and neural changes associated with growth of testis: (**a**) serum sex steroids (nmol/L) (AS—androstenedione; 4-AS-11B—4-Androsten-11B-OL3-17-dione; 4-AS-17B—4-Androsten-17B-OL3-11B-dione; 4-AS-3-11—4-Androsten-3-11-17-trione; TS—testosterone; PS—progesterone); (**b**) pituitary gene expression (microarray) (fish ID are the same as in panel (**a**)). Data are log_2_-ER centered to the average in all samples.

Serum levels of five steroid hormones (androstenedione, 4-androsten-11B-OL3, 4-androsten-17B-OL3, 4-androsten-3-11-17-trione, and testosterone) showed a strong correlation with GSI: Pearson r = 0.83 ± 0.05 (SD). With the exception of fish 160 (GSI 0.031%), serum levels of these hormones were relatively similar in the GSI range from 0.01 to 0.08% and increased 7.5–17.5-fold in the six fish with GSI ≥ 0.23% (Figure 2a, Supplementary Figure S1). Serum progesterone showed a significant (*t*-test, *p* = 0.04)—albeit smaller—increase in this group, while the serum levels of five steroid hormones (cortisol, 11-deoxycortisol, 21-deoxycortisol, corticosterone, and 4-Androsten-11B-17B-diol-3one) were not associated with the relative size of the testes. Microarray analysis of the pituitary gland revealed changes in the expression of genes involved in the endocrine control of maturation and indicated modification of tissue structure. In addition to fish 160, marked by high levels of serum steroids, fish 250 with GSI 0.043 showed expression profiles similar to those of fish that showed signs of sexual maturation (Figure 2b). This individual had enlarged germinative compartments with actively dividing cells (Table 1). Increased pituitary expression and serum levels of gonadotropins are strongly associated with sexual maturation of salmon [27]. *Nrob1* (*dax1*) is a nuclear receptor that plays a key role in testes development and male fertility [28]. *Aromatase* or *cyp19a1*, *hydroxysteroid* (*17-beta*) *3*, and *3 beta-hydroxysteroid dehydrogenases* catalyze the biosynthesis and conversion of sex steroids [29,30], and the progesterone receptor can be involved in feedback control of steroidogenesis. Somatolactin levels increase during salmon maturation and are closely correlated with 11-ketotestosterone. Somatolactin stimulates steroidogenesis in salmon testes in vitro, although its activity is considerably lower compared to gonadotropin [31]. In addition to endocrine regulators of maturation, we observed increased expression of hormones that control smooth muscle contraction (*protachykinin*, two paralogs) and behavior (precursor protein for opioid neuropeptides *prodynorphin*). Prodynorphin is also linked to the pubertal maturation of the reproductive neurosecretory axis, alongside factors such as kisspeptin and its receptor [32]. It seems to play a role in the timing of puberty onset in female pigs in the context of the hypothalamic-pituitary–gonadal axis, which is crucial for the initiation of puberty and the release of gonadotropins such as LH and FSH. The significant structural and functional rearrangement of the pituitary gland is indicated by the upregulation of the genes involved in synaptic transmission (*cholinergic receptor*, *LCR transmembrane neuronal protein 1*, and *synaptophysin*) and the differentiation and maintenance of neurons (*neuritin* and *neurofilament proteins*).

### 3.3. Testis Transcriptome

The data analysis of testicular gene expression was determined by the relative size of testes, with groups with GSI < 0.08% and GSI ≥ 0.23% (respectively, 14 and 6 fish). The latter group, which showed a marked concurrent increase in the expression of pituitary hormones, serum steroids, advanced germ cell development in testes, and increased GSI, is referred to as pubertal/maturing—as opposed to immature—salmon. For the interpretation of data, it is necessary to note that the observed differences could be caused by the disproportional growth of testicular compartments, the regulation of transcription, or their combination. The relative abundance of transcripts of genes with high and low expression in the germinative compartment increased or decreased, even in the absence of transcriptional changes, due to the increase in its proportion. Gene expression differences are commonly presented as up- and downregulation. Here, differential expression was assessed as in all our microarray analyses, but we prefer to use the terms “high and low specific activity” in pubertal testes (HSA and LSA) instead of, respectively, up- and downregulation. In total, 7792 genes showed a difference between the pubertal and immature testes, and gonad-specific genes (GSG) accounted for 10.2% (Table 2). The proportion of GSG among HSA was 3.3 times higher than that of LSA.

Functional groups with coordinated expression changes are searched by significant difference of mean log_2_-ER from zero baseline. Four large groups (from 38 to 239 genes) with high specific activity in pubertal testes are clearly associated with sexual maturation, which involves rapid cell division and DNA repair as part of meiosis, rearrangement of chromosomes, and the development of cilia, i.e., flagella of the spermatozoa (Table 3); most of these genes are gonad-specific. The composition of functional groups among LSA genes indicates considerable complexity of the interstitial compartment. Genes with decreased activity included genes of innate antiviral responses (211), lymphocyte-specific genes (211), regulators of differentiation (255), genes of nervous system (200), and genes encoding proteins of extracellular matrix (101). The greatest 8.2-fold difference was seen in immunoglobulins suggesting a potential depletion of B cells.

Developmental changes were found in 71 GSG with high activity in both testes and ovaries, 90% were among LSA, and some of them are known to be involved in female reproduction (Table 4). *P27kip1*- and chromatin-condensing *banf* control ovarian development [33,34]. *Cpebp1* regulates cytoplasmic polyadenylation and translation of mRNA during oocyte maturation, and *lsm14* is essential for oocyte meiosis [35]. *Ovostatins* (eight LSA genes) are protease inhibitors identified in eggs [36], and *fetuin* is a protease inhibitor that prevents premature hardening of the zona pellucida before fertilisation [37]. *Zp* glycoproteins (five and four LSA genes from two families) are components of an extracellular matrix surrounding oocytes that mediates sperm binding and prevents post-fertilization polyspermy.

GSC, which decreased specific activity in puberty, included several of the most studied and well-characterized regulators of reproduction (Table 5). *Dnd* [38], *nodal* [39], and *zglp1* [40] are essential for differentiation and maintenance of GC, whereas *nanos* controls the sexual differentiation of germ cells by promoting the male fate [41,42]. *Foxl2* plays a key role in the development of mammalian ovaries [43,44]; however, our transcriptome database shows a higher expression of the Atlantic salmon gene in testes. *Amh* is highly expressed by Sertoli cells from early fetal life to puberty when it is downregulated by male sex steroids [45]. This hormone inhibits the proliferation and differentiation of germ cells, as well as steroidogenesis in both sexes [46]. *Gsdf* is a fish-specific gene expressed mainly in Sertoli cells, which is involved in germ cell proliferation and male sex determination [47,48]. Another fish-specific gene that evolved from an immune regulator *irf9* determines male sex by arresting ovarian development [49]. *Inhibins* are antagonists of follicle-stimulating hormone (fsh) that are expressed in the interstitial compartment of salmonid testis [50]. The reduced responsiveness of the pubertal testes to external endocrine and neural signals was indicated by the decreased specific activity of genes that encode receptors for fsh, androgen, progesterone, and neuro mediators. Several GSG genes encoding hormones known for their association with maturation showed high specific activity in pubertal testes. Gonadal glycoprotein hormone alpha forms dimers with various beta subunits to activate hormone receptors [51]. The presence of non-hypothalamic *gnrh* in fish is known, but its function remains unclear [52]. In Atlantic salmon, expression of this gene is highest in the ovary, followed by the testis, brain, blood, and pituitary. *Boule* is highly expressed in male, but not in female gonads [5]. The transcription factor *e2f4* is essential for the development of the male reproductive system [53]. *Sfmbt1* can be involved in spermatogenesis as a regulator of the expression of canonical *histone* genes [54]. Transcription factor *sox-30* and transcription regulator *tdrd12* are located in spermatocytes and spermatids [55,56].

LSA in pubertal testes prevailed among genes encoding proteins of steroid metabolism (36 of 43 genes). Nine genes, including eight LSA, are involved in steroid hormone biosynthesis (Table 6). Mapping to the KEGG pathway (https://www.kegg.jp/kegg/mapper/assign_ko.html, Supplementary Figure S2) showed that these genes control all routes from cholesterol to hormones, and notably, only four of them are gonad-specific. Apart from testes, steroid hormones can be produced in other fish organs, including head, kidney, liver, intestine, and adipose tissue [57]; however, we are unaware of published data on the biosynthesis of sex steroids outside gonads.

The greatest specific activity in the pubertal testis was observed in genes directly involved in spermatogenesis; this group includes a total of 95 genes associated with flagella and motility, 34 genes with roles in meiosis, and 22 genes with various functions that do not fit into these two categories. A selection of the 25 genes with the highest activity within these categories is shown in Table 7. *Morn* is an uncharacterized sperm-specific protein [58]. *Slc9a10* maintains pH in spermatozoa [58] and *tcga10* is an essential component of spermatozoan cytoskeleton [59].

## 4. Discussion

This study addressed the relationship between gonadal growth and development in male Atlantic salmon with the aim of learning more about the regulation of these processes and identifying master genes. The results showed that the increase in the relative size of the testes in the interval of 0.01–0.08% was not associated with changes in the transcriptome. The structure of the tissue remained the same, although it grew many times faster than other tissues and the body, and distinct transcriptomic changes did not occur until advanced testis maturation (GSI of 0.2% and presence of SPB cells) was observed together with increased expression of pituitary sex hormones and sex steroids in serum. Testis transcriptome data are organized according to GSI because all fish with GSI less than 0.08% and greater than 0.23% had GE profiles characteristic of immature and mature testes, respectively. Based on plasma hormone levels and pituitary gene expression, fish 160 and 250 may be classified as sexually maturing, although in terms of the histological structure of the testes, they are much closer to immature males. This is most likely due to a delay between endocrine regulation and the onset of terminal spermatogenesis. Transcriptome changes indicated immature testis as a complex multifunctional tissue with high potential for different developmental scenarios, including angiogenesis, bone, cartilage, and nervous tissue formation. LSA included 314 genes that regulate differentiation and 84 growth factors and related genes. The immune activity of Atlantic salmon testes is relatively low, although higher than that in skeletal muscle or the brain [24]. However, sexual maturation was associated with a decrease in the specific activity of many immune pathways and functional groups in the testis, with B cells being the most affected. In European eels (*Anguilla anguilla*), several genes related to the immune system were downregulated in the brains of sexually mature males, and this may indicate the re-allocation of resources during maturation [60]. The available number of samples and techniques used in histology were insufficient to determine the precise threshold for the transition from hypertrophic growth to sexual maturation, and this decision point may vary between individuals depending on conditions and genetics. The initiation and rate of male maturity may show large disparity within the same population of fish. Factors affecting the onset of sexual maturation and the threshold of puberty have been previously discussed in the same fish material elsewhere [25].

Gonadal sex differentiation becomes apparent in female Atlantic salmon at 1.5–2 months post-hatching. Significant sex-related differences in testes gene expression can be found earlier, although testes seem to remain in an undifferentiated state for a longer time. Our transcriptomic data indicate that marked masculinization occurs during sexual maturation, as suggested by the decrease in transcripts of genes involved in oogenesis and the fertilization of oocytes. It should be noted that the histological analysis detected oocytes in 15% of the males, although only in fish with GSI < 0.08%. The occurrence and similar abundance of early previtellogenic oocytes in male testes has previously been observed in Atlantic salmon and is considered an intersexual state [61]. Thus, our data could be influenced by this phenomenon. The intersex condition in salmonids has been linked to the exposure or administration of endocrine-disrupting compounds, such as estrogenic chemicals [62]. Juvenile salmonids are sensitive to hormonal treatment, and this is used for sex reversal and the production of all-female salmon stocks in aquaculture [63]. We have no information about the potential presence of endocrine-disrupting compounds in the feed or water during the present study.

While pituitary gene expression meets expectations, the transcriptome profile of the testes may seem counterintuitive since reductions were observed in many genes involved in maturation, such as sex steroid biosynthetic enzymes and receptors for sex hormones. Regulation of gene transcription was probably masked by disproportionate growth, which increased the relative abundance of transcripts located predominantly or exclusively in the germinal compartment. Genes were selected with a 1.75-fold cutoff. Many genes showed a much larger difference, with the highest value being 207-fold (*proteoglycan 4*). Among the genes with a known specific role in salmon reproduction, *amh* (25-fold) and *irf9-like* (73-fold) were the most affected. Expression profiles were influenced by changes in tissue structure and transcriptional regulation, and we are unable to dissect and evaluate the contribution of these factors. Low specific activity means that gene expression might increase relative to the whole body, but it lagged behind in relation to testicular growth. Therefore, regardless of whether the transcription of hormone and neurotransmitter receptors was downregulated or not, the sensitivity of pubertal testes to external signals decreased, as did the specific activity of genes maintaining the GC population. The fish used in this study were exposed to different light conditions [25], which affected the proportion of mature males in the experimental groups. However, after the decision point, all testicles were the same. The light regime influences the likelihood of maturation entering, but once it has begun, the process becomes autonomous. Given the low specific activity of genes of steroid metabolism, one may ask whether the pubertal testis is the sole or primary site of sex steroid biosynthesis. In general, the observed changes indicated functional simplification of the testes and polarization towards reproduction. Many functional groups were present among LSA, whereas HSA included only four groups, but with a large number of gonad-specific genes. Transcriptome data highlighted many uncharacterized Atlantic salmon genes involved in meiosis and sperm motility and also provided rich information on the distribution of gonad-specific genes between the interstitial and germinal compartments; the results in Supplementary Table S1 can be a useful source of information.

Transcriptome analyses aim to find unknown regulators of Atlantic salmon male reproduction and gene markers for precocious puberty. Sexual maturation begins with the production and secretion of pituitary sex hormones, followed by an increase in sex steroid levels and changes in the structure of the testes. We do not know if the onset of puberty is completely controlled by the pituitary gland, or whether the final decision lies with internal regulators within the testes. To identify triggers, it is necessary to examine fish at the transition from endocrine stimulation to tissue changes, such as the hypertrophy of the germinal compartment, meiosis, and sperm differentiation. This study was carried out on twenty male salmon, and two fish could fulfill this requirement. Detailed investigation of such fish may be one of the most interesting topics for future research. As an illustration, we picked 35 genes with increased expression in six pubertal males and fish 160, which had high serum levels of sex steroids, but not in fish 180, which only showed changes in pituitary hormones. Half of these genes are involved in chromosome maintenance, the cell cycle, and regulation of transcription, and eight genes are gonad-specific (Supplementary Table S2). The available information is not sufficient to consider these genes to be possible triggers, but the accumulation of data will help us to identify the master genes for male puberty in Atlantic salmon.

## 5. Conclusions

The study identified major gene expression changes associated with testis growth and differentiation in Atlantic salmon. Once initiated, maturation appears to become largely autonomous, and sensitivity to external regulators weakens. Spermatogenesis predominates, while various developmental and immune processes are suppressed. The role of many genes involved in reproduction has been confirmed, and uncharacterized genes that may regulate puberty have been identified.

## Figures and Tables

**Table 1:** Overview of testes histology with developmental stages.

FISH ID	Stage (I–V)/Most Advanced Cell Type	Stage (I–V)/Dominating Germ Cell Type	Size of Germinative Compartments	Observations
188	I/SPA	I/SPA	Small (<80 μm)	
233	I/SPA	I/SPA	Small (<80 μm)	
240	I/SPA	I/SPA	Small (<80 μm)	
176	I/SPA	I/SPA	Small (<80 μm)	
193	I/SPA	I/SPA	Small (<80 μm)	
203	I/SPA	I/SPA	Medium (80–140 μm)	tubules, high cell division
236	I/SPA	I/SPA	Small (<80 μm)	
160	I/SPA	I/SPA	Medium (80–140 μm)	tubules, high cell division
198	I/SPA	I/SPA	Small (<80 μm)	oocytes
255	I/SPA	I/SPA	Small (<80 μm)	oocytes
250	I/SPA	I/SPA	Small (<80 μm)	
221	I/SPA	I/SPA	Small (<80 μm)	oocytes
226	I/SPA	I/SPA	Small (<80 μm)	
180	I/SPA	I/SPA	Small (<80 μm)	
184	IV/SPT	II/SPB	Medium (80–140 μm)	
190	IV/SPT	III/SPC	Large (43–500 μm)	
213	III/SC	III/SPC	Large (43–500 μm)	
245	V/SZ	III/SPC	Large (43–500 μm)	
211	V/SZ	III/SPC	Large (43–500 μm)	
202	V/SZ	III/SPC	Large (43–500 μm)	

SPA, spermatogonia A undifferentiated and differentiated; SPB, spermatogonia B; SPC, spermatocytes; SPT, spermatids; SZ, spermatozoa.

**Table 2:** Numbers of genes with high (HSA) and low specific activity (LSA) in pubertal testes compared to immature testes.

Genes	All	Gonad-Specific	% Gonad-Specific
HSA	3524	584	16.6
LSA	4268	214	5.0
Total	7792	798	10.2

**Table 3:** Functional groups of genes (STARS annotation) with increased and decreased activity in pubertal testes. Numbers of gonad-specific genes with low and high specific activity (LSA and HSA) are indicated. In this and subsequent tables, gene expression data are pubertal-to-immature testes folds.

Functional Group	Genes Number	Gonad-Specific LSA/HSA	Mean Fold
Cell cycle	239	5/70	1.8
Chromosome	212	1/58	2.1
DNA repair	38	0/16	2.7
Cilia	83	1/59	3.8
Antigen presentation	84	0/0	−4.3
Chemokine	20	0/1	−4.1
Antiviral	211	0/0	−3.8
Complement	40	2/1	−4.0
Immunglobulins	40	0/0	−8.2
Lymphocyte	111	0/0	−3.4
Secreted proteins	25	7/1	−3.1
Endocrine	70	11/4	−2.2
Growth factors	84	4/3	−2.6
Differentiation	255	9/8	−2.2
Differetiation-hox	59	9/2	−2.5
Angiogenesis	34	0/1	−3.1
Bone, cartilage	28	3/2	−2.8
Neural	200	10/3	−2.2
Epithelium	26	0/3	−2.5
Extracellular matrix	101	2/7	−2.6
Collagens	66	3/1	−2.5

**Table 4:** Gonad-specific genes (males and females) with low specific activity (LSA) in pubertal testes. Genes with confirmed roles in oogenesis are italicized.

Gene	Fold
Apopolysialoglycoprotein	−5.5
*Barrier-to-autointegration factor (banf)*	−4.4
*Cyclin-dependent kinase inhibitor 1B-like (p27kip1)*	−7.8
*Cytoplasmic polyadenylation element-binding 1 (cpebp1)*	−3.6
*Fetuin-B-like*	−4.5
Glucosamine-6-phosphate deaminase	−3.3
Late histone H2A.2.2-like	−2.1
*LIM homeobox 8a*	−3.4
*Oocyte-specific histone RNA stem-loop-binding 2*	−2.1
*Ovostatin-like (8 genes)*	−4.7
*P43 5S RNA-binding protein-like (2 genes)*	−4.2
*LSM14 homolog B-like (lsm14b)*	−2.0
*Zona pellucida sperm-binding 3-like (zp3, 5 genes)*	−13.5
*Zona pellucida sperm-binding 4-like (zp4, 4 genes)*	−6.8

**Table 5:** Genes with known or predicted roles in the regulation of reproduction.

Gene	Fold
Dead end protein 1 (dnd)	−2.5
Nanos homolog 2-like	−6.8
GATA-type zinc finger protein 1 (zglp1)	−3.5
Nodal homolog (2 genes)	−13.2
Anti-mullerian hormone (amh)	−25.3
Gonadal somatic cell-derived factor (gsdf)	−5.7
Interferon regulatory factor 9 (irf9)	−73.2
Forkhead box protein L2-like (foxl2)	−5.4
LIM homeobox 2b	−2.0
LIM homeobox 8a	−3.4
Inhibin beta A chain (2 genes)	−6.5
Inhibin, alpha	−4.1
Androgen receptor	−4.4
Progesterone receptor	−4.9
Follicle stimulating hormone receptor	−7.0
GABA receptor subunit alpha-2	−20.9
Glutamate receptor ionotropic, NMDA 2C	−8.6
Metabotropic glutamate receptor 8-like	−3.3
Glycoprotein hormone alpha-2	4.6
E2F transcription factor 4	2.9
Furin-like	3.2
Progonadoliberin-1 isoform 2	6.0
Protein boule-like	3.7
Scm-like with four MBT protein 1 (sfmbt1)	2.2
Transcription factor SOX-30	6.8
Tudor domain containing 12 (tdrd12)	3.1

**Table 6:** Genes involved in biosynthesis of steroids. Gonad-specific genes are italicized.

Gene	Fold
25-hydroxycholesterol 7-alpha-hydroxylase	−2.4
*3 beta-hydroxysteroid dehydrogenase*	−4.2
3-oxo-5-beta-steroid 4-dehydrogenase	−4.0
*Corticosteroid 11-beta-dehydrogenase isozyme 2*	−5.0
Cytochrome P450 1B1-like	−2.3
*Cytochrome P450, family 17A1*	−5.4
Dehydrogenase/reductase SDR family 11	−2.8
*Hydroxysteroid 11-beta dehydrogenase 2*	−6.0
Hydroxysteroid 11-beta-dehydrogenase 1-like	4.2

**Table 7:** Genes involved in spermatogenesis: 25 genes with top HSA in pubertal testes.

Gene	Fold
*Flagella, motility*	
Cilia and flagella associated protein 61	7.9
Dynein heavy chain 10, axonemal	8.6
Dynein light chain 4, axonemal	9.4
Male germ cell-associated kinase	7.0
Primary cilia formation	8.3
Radial spoke head protein 6 homolog A	7.9
Ropporin-1-like	8.2
Sperm associated antigen 16	10.1
Sperm flagellar protein 2-like	9.1
Sperm-associated antigen 6	8.3
WD repeat-containing protein 96	7.4
*Meiosis*	
HORMA domain-containing protein 1	17.0
Meiotic recombination protein DMC1/LIM15	12.0
Meiotic recombination protein REC114-like	13.3
Meiotic recombination protein SPO11	7.1
Minichromosome maintenance domain 2	7.1
SHC SH2-domain binding protein 1-like	15.3
Spermatogenesis-associated protein 22-like	7.7
Synaptonemal complex central element 1	11.1
Synaptonemal complex protein 3 like	8.3
Zebrafish testis-expressed 38	7.0
Zinc finger protein 541-like	7.4
*Various roles*	
MORN repeat-containing protein (*morn*)	7.5
Sodium/hydrogen exchanger 10-like (*slc9a10*)	7.6
Testis-specific gene 10 protein	6.6

## Data Availability

Microarray data were submitted to NCBI Geo Omnibus (GSE263627).

## Supplementary Materials

The following supporting information can be downloaded at https://www.mdpi.com/article/10.3390/cimb46060319/s1: Table S1: Genes with expression differences between immature and pubertal testes; Table S2: Genes with earliest response to sex steroids; Figure S1: Normalized plasma levels of sex steroids (each value divided by the mean hormone level) versus log_2_-GSI; Figure S2: Steroid hormone biosynthesis (KEGG pathway). Differentially expressed genes are highlighted in red.
