# Supplementary figures and images for: Endocrine and Transcriptome Changes Associated with Testicular Growth and Differentiation in Atlantic Salmon (Salmo salar L.)
Source: Curr Issues Mol Biol. 2024 May 27;46(6):5337–51. doi: 10.3390/cimb46060319 (PMC11202266; doi:10.3390/cimb46060319)

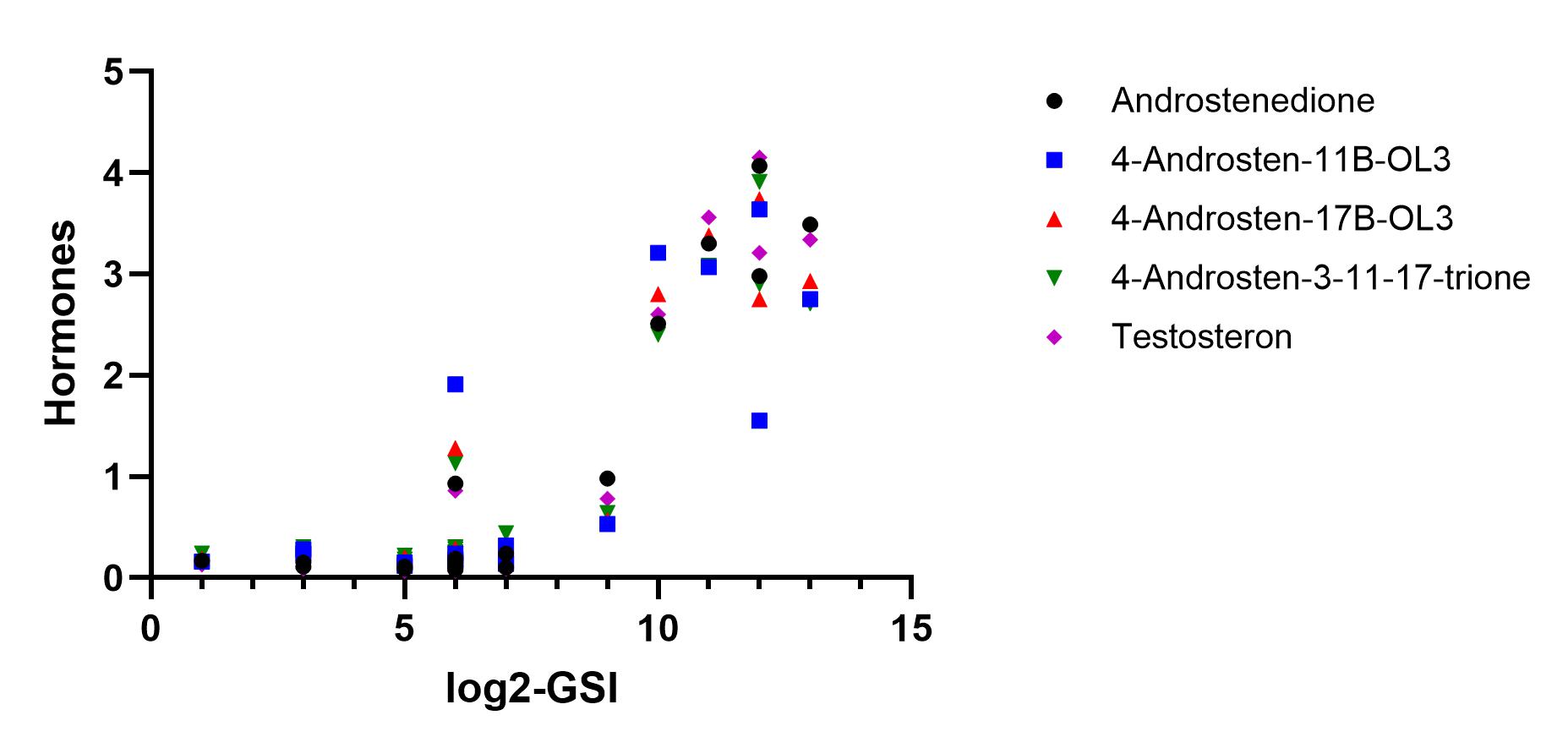

Supplement: Supplementary file 1 [file cimb-46-00319-s001.zip › cimb-2989970-supplementary/Supplement figure 1.jpg]

Change pathway type

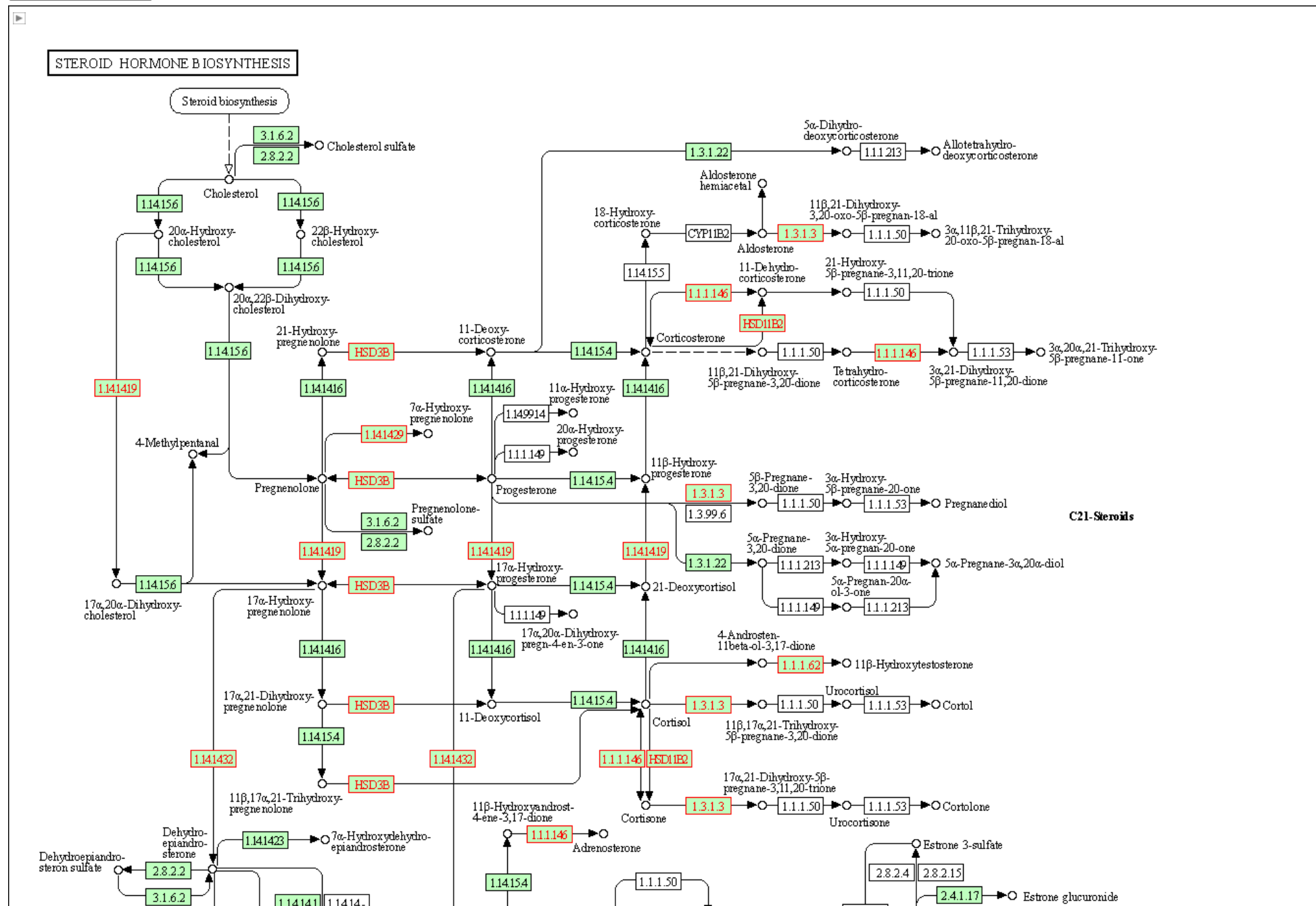

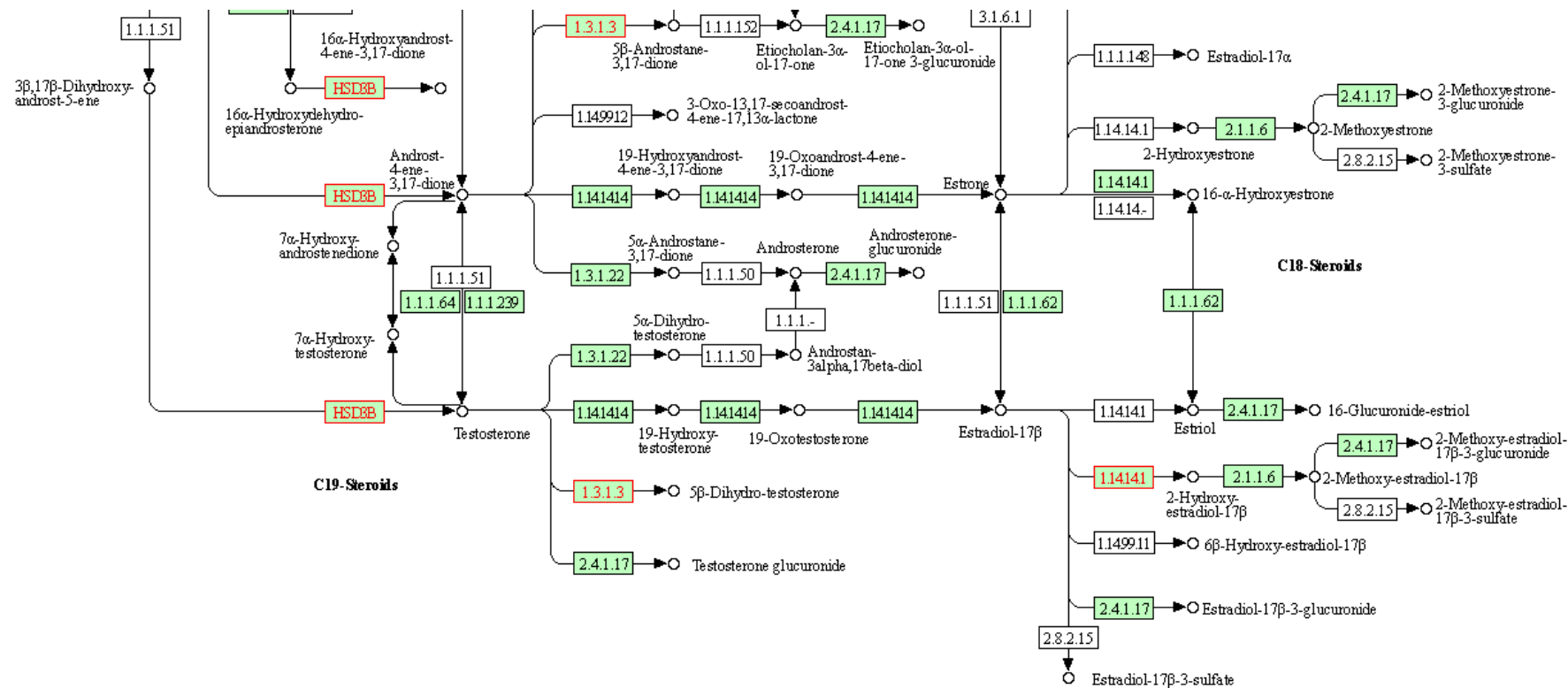

00140 10/11/22  
(c) Kanehisa Laboratories

Supplement: Supplementary file 1 [file cimb-46-00319-s001.zip › cimb-2989970-supplementary/Supplement figure 2..pdf]
